# Discharged Soldiers' Allowances

**Published:** 1916-10-21

**Authors:** 


### NATIONAL INSURANCE ACT DEVELOPMENTS.

### Discharged Soldiers' Allowances.

An article contributed by a correspondent appeared in the *Morning Post* under the above title on September 28. In it the writer freely criticises the Insurance Commissioners and the "insurers" (meaning presumably the approved societies) on account of the difficulties which are stated to be experienced by discharged soldiers in obtaining benefits that are due to them under the National Insurance Acts. Although the article contains a good deal that is irrelevant to the subject of discharged soldiers' allowances, its criticisms are so forcibly expressed as to attract attention; so much so, in fact, that we have taken some pains to ascertain how far they are justified. The writer makes the startling statement, for instance, that there is "great waste of time and consequently of money spent in fending off payment of National Insurance benefits." This is a serious charge if it can be supported, and although the writer specifically states that he does not believe in the practice of quoting individual cases, he does actually give particulars of two—and only two—cases which may appear to justify his conclusions.

### The Asylum Case Difficulty.

The first case relates to a discharged soldier in an asylum, and the complaint is that while he is an inmate of that institution it is impossible to secure for his personal use the sickness benefit that is accruing to him. The reason for this appears in a remark which the writer makes in parenthesis—namely, that the member has no dependants. In these circumstances it is not the fault of the approved society of which he is a member, nor indeed of the Insurance Commissioners, that payment is withheld. If, in fact, it be the fault of anyone, it is Parliament that must take the blame for having enacted that the payment of sickness or disablement benefit shall in these circumstances be paid to the insured person on leaving the institution, unless an agreement has been made between the institution and the approved society for the treatment of the member or of its members generally. There were undoubtedly good reasons both for and against such an enactment, which were freely discussed at the time the matter was before Parliament, and into the merits of which we need not now enter. It need only be remarked that it is the old question of the non-recognition of hospital and institutional treatment under the National Insurance Acts which the writer of the article has re-discovered as adversely affecting the interests of discharged soldiers. There is, however, one point in the writer's statement which appears to us to be new—namely, his reference—twice repeated—to the necessity of making a claim within three months of discharge (presumably from the Army, and not from the institution). It is possible that the particular society to which the man belongs has a rule stipulating that claims for arrears of benefit cannot be entertained for a longer period than three months, but such a rule is unusual, and we have been unable to find any trace of a general instruction in which such a limit is imposed. But a rule of this description would be perfectly justifiable; for the proper administration of sickness benefits can only be attained when it is possible for the claims to be investigated, and the lapse of time would prejudice the evidence that could be produced in support of a claim. Moreover, it is laid down in the definition of sickness benefit in the principal Act that notice must be given of the incapacity for work. It would be unreasonable to expect approved societies or the Commissioners to appoint representatives merely to hunt out cases which have a right to claim benefits but have not exercised those rights.

The writer of the article is not, therefore, justified in basing his conclusion that the approved societies and the Insurance Commissioners are desirous of "fending off payments" on the evidence of the asylum cases to which he refers.

### Formalities may be Necessary.

The delay in the second case mentioned by the writer was due in the first instance, on his own showing, to a discrepancy between the ages given on the discharge papers and that shown by the society's own records. Both statements of age were due, directly or indirectly, to the insured man himself, and the society was unable without further evidence to determine which of the two was correct. As, in the particular case, the question of age was material in determining the rate of benefit, in view of the fact that the discharge papers showed him to be a minor, the society had no alternative but to defer the admittance of the claim in order that it might satisfy statutory requirements. Again, there was further delay owing to the fact that the man had not produced the requisite evidence of his discharge. It is urged by the writer that the production of such evidence is a pure formality that might have been dispensed with, but though a formality it is one that is very necessary to prevent the abuse of benefits. No sickness or disablement benefit is payable so long as the man is in the Army, hence the necessity for the evidence of his discharge. There ought, however, to have been no difficulty in the matter, for the man should have had in his possession Form 01845, which is handed or sent to each man on his discharge by the Army authorities, and which, by the instructions of the Insurance Commissioners, is required to be produced as evidence before approved societies can grant benefits to discharged soldiers. The subsequent waste of time, to which the writer was unfortunately put, was due to misdirected zeal on behalf of the man; for he called at the offices of the Insurance Commission in Buckingham Gate, whereas he should have called at the offices at Maida Hill, where for the time being all matters relating to the insurance of soldiers are being dealt with.

Lack of Interest in Insurance.

We have frequently found it necessary to criticise the administration of National Health Insurance, and the unbusinesslike methods of the Commissioners, but it is only fair to state that from our own experience and from inquiries made in reliable quarters there is no desire either on the part of the approved societies or of the Commissioners to "fend off" payments that are due. On the contrary, the Commissioners, acting through the central staff, are as keen on seeing that insured persons secure their rights under the Acts as they are in seeing that the approved societies do not make payments improperly.

One further matter only remains to be added, arising from the article. The writer states that "cases are not uncommon where men have torn up their papers rather than go through the weariness of again and again answering questions already answered." This statement is applied by the writer to the filling in of claim papers. It would have been more correct if he had stated that many men—possibly running into hundreds of thousands—took so little interest in their insurances that they failed to notify their approved societies of their enlistment, in spite of the fact that a special post-card is supplied to each man for the purpose. It must not be forgotten that the large approved societies cannot possibly keep records of their members if the latter do not comply with such simple requirements as the notification of enlistment. In normal times a case would be traced at the central office of any of the large approved societies by reference to the membership number, the name of the member being of minor importance and used only to see that the correct case was being dealt with. But so widespread has been the disregard of giving notice of enlistment, and so little care has been taken to keep any record of the insurances, that at least one of the large approved societies has perforce had to set up at considerable expense a special alphabetical index of its members for the purpose of tracing such cases. We have even heard of men who had forgotten the name of their society.

